# A Rare Paraneoplastic Syndrome of Lung Cancer

**DOI:** 10.1155/2020/7891325

**Published:** 2020-06-21

**Authors:** R. Azzeddine, L. Herrak, A. Rhanim, A. Jniene, M. Elftouh, L. Achachi

**Affiliations:** Department of Pulmonology, IbnSina Hospital, Rabat, Morocco

## Abstract

Achalasia is a neurodegenerative motor disease of the esophagus resulting mainly from a loss of function of the lower esophageal sphincter, the pathophysiology of which is still poorly understood. Its incidence is rare—it is 1.6 per 100,000—and its occurrence in the context of paraneoplastic syndrome has been rarely described in the literature. We report a rare case of paraneoplastic achalasia associated with lung cancer.

## 1. Introduction

Achalasia is a motor disorder of the esophagus characterized by a peristalsis and incomplete lower esophageal sphincter relaxation without evidence of mechanical obstruction. Diagnosis is established by esophageal manometry and may be supported by findings on barium esophagogram [1], its incidence is 1.6 per 100,000 [2]. Paraneoplastic achalasia is a very rare entity, and we report a case of epidermoid bronchopulmonary carcinoma manifesting as achalasia.

## 2. Case Report

We report the case of a 70-year-old man who was neither treated for tuberculosis nor has had any recent tuberculosis contagion, and with no notable pathological history or chronic smoking. The patient has reported a productive cough for 2 months, with mucous sputum associated with stage III of mMRC dyspnea, without hemoptysis or chest pain. Moreover, for 1 month he had liquid dysphagia associated with regurgitations, in the context of apyrexia and deterioration of the general state.

Clinical examination finds a conscious patient, eupneic at rest, saO_2_ = 92% at ambient air, with a pleuropulmonary examination without particularities.

**Figure 1:**
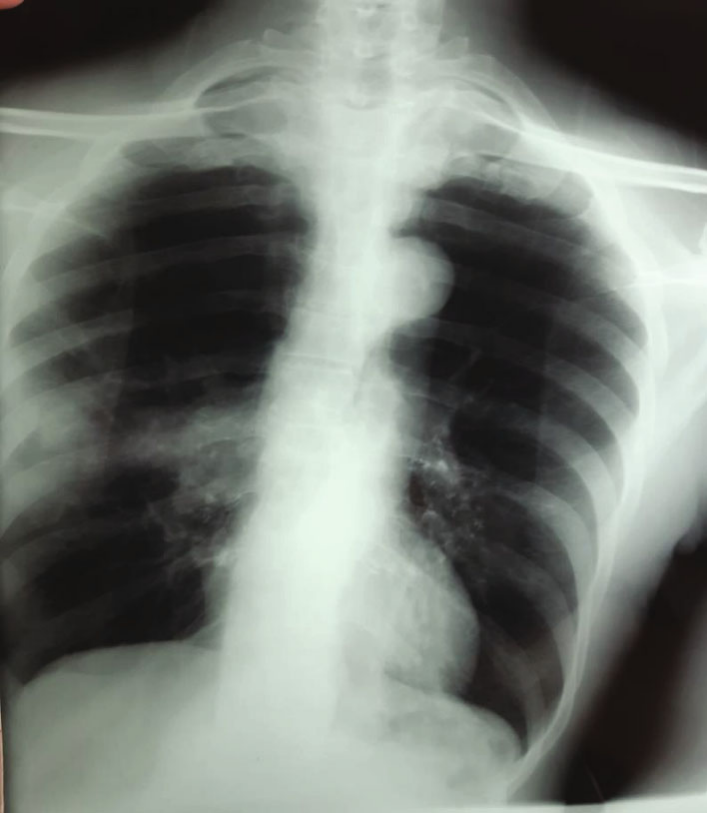
The posteroanterior chest roentgenogram showed homogeneous right hilar opacity with peripheral nodular opacity with spiculated contours.

**Figure 2:**
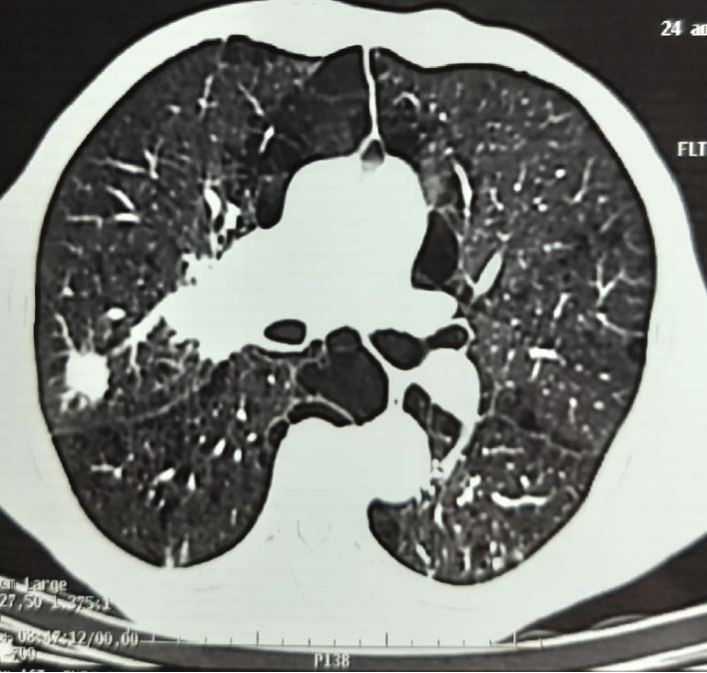
The thoracic computed tomography (CT) showed a right hilar tumoral process extended to the posterior segment of the right upper lobe of irregular and spiculated contours, associated with a spiculated nodule of the right upper lobe.

The posteroanterior chest roentgenogram showed homogeneous right hilar opacity with peripheral nodular opacity with speculated contours (Figure 1). The thoracic computed tomography (CT) showed a right hilar tumoral process, extended to the posterior segment of the right upper lobe with irregular and spiculated contours, measuring 42 × 32 × 25 mm without contact with the esophagus, associated with a spiculated nodule of the right upper lobe measuring 20 × 19 mm, without lymphadenopathy mediastinal (Figure 2).

A bronchial fibroscopy was made objectifying a budding mucosa of the right upper lobar bronchus whose biopsy concluded from an epidermoid carcinoma.

As part of the exploration of dysphagia, an oesophagoscopy was normal and an achalasia was evoked and confirmed by esophageal manometry.

Due to the significant alteration of the patient, the patient died before the initiation of treatment.

## 3. Discussion

Paraneoplastic syndromes refer to the remote effects associated with malignancies which are unrelated to direct tumor invasion or metastases. These may occur before the cancer is diagnosed and can be independent in their severity to the stage of the primary tumor. Paraneoplastic syndromes are most commonly associated with lung cancer, reported in approximately 10% of the cases. Endocrine syndromes, particularly the syndrome of inappropriate ADH secretion (SIADH) and humoral hypercalcemia of malignancy (HHM), are the most common paraneoplastic syndromes seen in lung cancer and are related to the histologic type of cancer [3].

Hypercalcemia has been reported in 2–6% of lung cancer cases; when associated with PTHrP (parathyroid hormone-related protein) production, it is referred to as HHM. Of the four mechanisms of hypercalcemia secondary to HHM (secretion of PTHrP, parathyroid hormone, 1-25 dihydroxy vitamin D, or granulocyte colony stimulating factor), secretion of parathyroid hormone-related protein is the most common in lung cancer. It is associated with a poor prognosis [3].

SIADH represents a state of euvolemic hypoosmolar hyponatremia, which in the case of lung cancer is secondary to ectopic ADH production; 10–45% of small-cell lung cancers (compared to 1% of non-small-cell lung cancers) can produce ectopic ADH resulting in excessive urinary sodium excretion. Hypothyroidism, volume depletion, and adrenal insufficiency should be excluded [3].

The secretion of ectopic growth hormone releasing hormone (GHRH) from malignant cells can manifest as acromegaly, and in the case of lung cancer, bronchial carcinoids and epidermoid carcinomas have been implicated; SCLC have been reported less frequently [3].

Venous thromboembolism (VTE) including DVT, PE, and superficial vein thrombosis occur in nearly 3% of lung cancer patients within the first 2 years of diagnosis. Patients with lung cancer have a 20-fold increase in the risk of VTE compared to the general population. NSCLC confers a higher VTE risk than SCLC, and adenocarcinomas are associated with a higher risk of VTE than squamous cell carcinoma. Distant metastases confer a fold increase in VTE compared to localized tumors. More so, tissue factor (TF), which initiates the coagulation cascade and cancer procoagulant, have an increased expression in lung cancer cells [3].

Paraneoplastic neurological syndromes (PNSs) are autoimmune in nature; unlike most paraneoplastic syndromes, they are independent of local tumor or metastatic effects. Onconeural antibodies appear to be central to the pathogenesis, though their absence does not preclude a diagnosis of PNS [3]. These antibodies are directed against tumor cells but can also target the nervous system (central and peripheral), resulting in the wide-ranging manifestations of PNS.

The first description of autonomic neuropathy linked to lung cancer dates back to 1975 [4], and it was not until 1985 that anti-Hu Ab were isolated, from patients suffering from paraneoplastic neuropathies [5] and in 1993 that the first association between lung cancer, pseudochronic intestinal obstruction, and anti-Hu Ab has been reported [6]. Since then, anti-Hu Ab have been identified in other digestive dysautonomies (achalasia, gastroparesis), as well as in multiple extradigestive neurological disorders [7]. These antibodies are directed against a family of proteins, which are specific to neurons in the central, peripheral, and enteric nervous systems. Although other antineuronal antibodies (anti-YO, anticalcic, and anticalcium) have been demonstrated during paraneoplastic syndromes affecting the digestive system, anti-HU are in the majority [8].

Fewer than a hundred cases of motor digestive damage of paraneoplastic origin have so far been described in the literature [9].

The most important series of digestive motor damage of paraneoplastic origin was reported in 1989 by Lucchinetti et al. [7]. Their report included 38 patients, out of which 19 had gastroparesis (50%), 8 had pseudochronic intestinal obstruction (POIC) (21%), and only four had achalasia (10%).

The most commonly associated neoplasia is lung cancer (81%), which perfectly matches the case of our patient.

The term “achalasia” derives from the Greek “khalasis” whose translation is “relaxation” [10]; it is a motor disorder of the esophagus characterized by a peristalsis and incomplete lower esophageal sphincter relaxation without evidence of mechanical obstruction that results from the degeneration of ganglion cells in the myenteric plexus of the esophageal body and the lower esophageal sphincter, leading to incomplete relaxation of the lower esophageal sphincter and a peristalsis in the distal esophagus [1].

As mentioned in the Introduction, the incidence is low—approximately 1.6 per 100,000 [1]—equally distributed between men and women and increasing with age, with a peak between 40 and 60 years [10].

Dysphagia to both solids and liquids is the defining symptom of achalasia (occurring in 90% of patients), and patients may compensate for dysphagia by eating slowly or performing various maneuvers while eating (e.g., raising the arms, lifting the neck, and arching the back) [1]. Paradoxical dysphagia (electively to liquids) is rare but strongly suggestive, as in the case of our patient.

Regurgitation of undigested food or saliva, with or without dysphagia, occurs in 76% to 91% of patients, especially during recumbency at night, which may result in aspiration or cough.

Symptoms of gastroesophageal reflux, such as substernal chest pain, epigastric pain, or heartburn, occur in approximately half of the patients; symptoms do not resolve with a trial of proton pump inhibitors. Other symptoms include odynophagia, halitosis, cough, hoarseness, wheezing, sore throat, and difficulty burping.

Achalasia results from the degeneration of ganglion cells in the myenteric plexus of the esophageal body and the lower esophageal sphincter, leading to an incomplete relaxation of the lower esophageal sphincter and a peristalsis in the distal esophagus. The exact cause of the degeneration leading to achalasia is unknown, but autoimmune, viral, or primary neurodegenerative processes are suspected [1].

Diagnosis is established by esophageal manometry and may be supported by findings on barium esophagogram [1].

There is no curative or preventive treatment for the degeneration of the myenteric esophageal plexus. The treatments available are aimed at reducing the pressure of the LES by rupture of the circular muscle fibers. The treatments traditionally proposed are endoscopic pneumatic dilation and laparoscopic Heller's myotomy. Endoscopic peroral oral myotomy is a technique currently practiced routinely in expert centers; it is effective and safe, and its exact place in the therapeutic management of achalasia remains to be defined [7].

## 4. Conclusion

Paraneoplastic syndromes are most commonly associated with lung cancer, though paraneoplastic achalasia is rarely described in the literature. Through our work, we report a rare paraneoplastic syndrome of lung cancer, which is achalasia, in order to focus on this entity and sensitize practicing physicians to seek an underlying neoplasia when treating achalasia. The exact pysiopathology of this entity is still poorly defined, and new ways of research must be explored in the future to better understand this rare association.
